# Influence of Traffic Input Data Quality on Road Noise Estimates Using the CNOSSOS-EU Method

**DOI:** 10.3390/s26030778

**Published:** 2026-01-23

**Authors:** Elena Ascari, Cătălin Andrei Neagoe, Mauro Cerchiai, Gaetano Licitra, Ana-Maria Mitu, Tudor Sireteanu, Daniel Cătălin Baldovin, Luca Fredianelli

**Affiliations:** 1 Institute of Chemical and Physical Processes of National Research Council, 56123 Pisa, Italy; 2 Institute of Solid Mechanics of the Romanian Academy, 70701 Bucharest, Romania; 3 Department of Strength of Materials, Faculty of Industrial Engineering and Robotics, National University of Science and Technology POLITEHNICA Bucharest, Splaiul Independentei 313, 060042 Bucharest, Romania

**Keywords:** road traffic noise, CNOSSOS-EU, traffic data collection, radar traffic counter, AI camera, Google API, noise modeling, environmental noise assessment, road traffic monitoring, strategic noise mapping

## Abstract

Accurate traffic input data are essential for reliable road noise mapping within the CNOSSOS-EU framework. However, European countries often rely on heterogeneous data sources and measurement practices, which may introduce uncertainties in noise estimates and reduce the comparability of results across regions. This study evaluates the performance of three traffic data collection methods, specifically microwave radar traffic counters, artificial intelligence-based cameras, and Google API-derived flows, in three representative test sites located in Italy and Romania. Traffic flows and vehicle category distributions obtained from each method were used as inputs for noise simulations, and predicted levels were compared with in situ noise measurements. A second analytical approach was developed to estimate short-term noise levels at a 10’ resolution by combining CNOSSOS-EU power models with propagation matrices computed using commercial sound propagation software. The results show that both radar counters and cameras provide reliable inputs for day/evening/night indicators, although counters may miss flows under complex traffic conditions, and cameras may overestimate counts at high volumes. Google API-derived flows perform well only when traffic exceeds approximately 150 vehicles per hour and when the traffic model is carefully calibrated. Manual counting confirmed that all three input data collection methods exhibit non-negligible traffic loss, which contributes to a systematic underestimation of simulated noise levels when using average flow-based modeling. Differences between methods become more pronounced when analyzing short time intervals rather than aggregated indicators. Overall, this study highlights the strengths and limitations of each data source and provides guidance on their appropriate use for road noise assessment and strategic mapping.

## 1. Introduction

Environmental noise has been recognized for decades as a major public health concern throughout Europe [1,2,3,4,5,6]. It is associated with cardiovascular and metabolic impacts, cognitive impairment in children, annoyance, and sleep disturbance, making it the second largest environmental cause of health problems after air pollution. According to the Environmental Noise in Europe 2025 report [7], more than 20% of Europeans are exposed to harmful transport noise levels above the thresholds defined by the Environmental Noise Directive (END) [8]. This proportion exceeds 30% when compared to the World Health Organization recommendations [9]. Road traffic is the dominant source of exposure, affecting an estimated 92 million people with a L_DEN_ greater than 55 dB and contributing to approximately 66.000 premature deaths and 50.000 new cases of cardiovascular disease each year. The associated health burden amounts to roughly 1.3 million disability adjusted life years lost annually, corresponding to an economic impact of about EUR 95.6 billion.

To address these challenges, the European Commission introduced the END in 2002, establishing the requirement for strategic noise mapping of major roads, railways, airports, and industrial sources [10,11,12,13,14]. Noise mapping relies on mathematical models that describe the propagation of sound in the environment. Since 2015, the official model for END implementation has been the CNOSSOS-EU method [15], which replaced the former interim NMPB-96 model [16]. CNOSSOS-EU became mandatory for all EU Member States in the fourth round of noise mapping in 2022.

Several studies have shown that CNOSSOS-EU tends to predict lower road traffic noise levels than NMPB-96 [17,18,19,20,21,22,23], with differences attributed to revised vehicle categories and updated formulations of absorption and diffraction [24]. While such offsets may not influence noise mitigation design, they play a critical role in exposure and health impact assessments that require accurate and validated estimates. The existing literature has investigated these discrepancies but has rarely addressed the influence of input data quality on CNOSSOS-EU predictions.

Noise modeling accuracy depends directly on the quality of the input data. Although widely recognized, this concept has been only partially explored in the context of CNOSSOS-EU. Previous analyses of traffic input quality were mostly limited to NMPB-96 [25] or based on idealized conditions described in technical guidelines. Moreover, there is increasing evidence that all commonly used traffic data collection systems exhibit residual detection losses, even when applied under controlled conditions. Such losses propagate through the noise modeling chain and tend to generate a systematic underestimation of simulated levels, particularly when average flows are used in place of detailed temporal profiles.

This study was carried out in the framework of a bilateral collaboration between the National Research Council of Italy and the Romanian Academy [26]. The aim was to assess the availability and reliability of traffic input data for CNOSSOS-EU implementation in Romania and to evaluate how different data collection methods influence noise predictions in real urban and peri-urban environments. The work focuses specifically on the variability introduced by the input data collection methods rather than on comparisons with the former interim model, even if this is also addressed in the framework of the collaboration.

Three traffic data acquisition techniques were analyzed, namely microwave radar traffic counters, artificial intelligence-based cameras, and Google API-derived flows that were processed through Python 3.12/3.13 scripts. These methods range from standard commercial products, such as radar traffic counters, to more innovative technologies: AI cameras are being developed by different scholars for the purpose of noise estimates [27,28] or to identify noisy vehicles [29], and are now also available as commercial products for this latter purpose; APIs have been used to derive traffic information by several scholars [30,31,32,33], but only in a few studies were noise estimates included [34,35]. Their outputs were compared in terms of total flows, vehicle category distributions, and short-term variability. These datasets were then used as inputs for noise simulations, and the resulting noise levels were compared with in situ acoustic measurements. In addition to the quantitative analyses, this study incorporates qualitative Strengths, Weaknesses, Opportunities, and Threats (SWOT) analysis. This framework is widely used to synthesize the operational performance of alternative technologies and provides a practical assessment of their applicability, benefits, and limitations in real monitoring scenarios. By combining quantitative evaluation and qualitative synthesis, this study provides a comprehensive assessment of how input quality affects CNOSSOS-EU noise predictions and offers guidance for future applications in strategic noise mapping.

## 2. Materials and Methods

**Figure 1:**
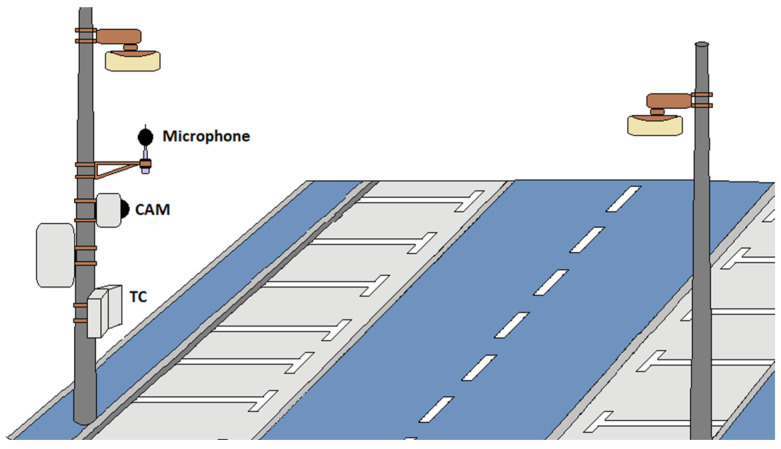
Example of a noise monitoring station equipped with the microwave radar traffic counter (TC) and the artificial intelligence-based camera (CAM).

The present study investigates how different traffic data collection methods influence road traffic noise estimates produced with the CNOSSOS-EU model. Three input data collection methods were analyzed: microwave radar traffic counters (TC) produced by via traffic controlling gmbh (Leverkusen, Germany), artificial intelligence-based cameras (CAM) [28] provided by IPOOL SRL (Pistoia, Italy), and API-derived flows (API) [35] provided by Google LCC (Mountain View, CA, USA). Each dataset was processed to obtain vehicle flows, vehicle category distributions, and average speeds. Two complementary modeling strategies were adopted. The first approach follows the standard Environmental Noise Directive procedure, using average day, evening, and night flows to compute noise indicators. For each country, the specific time definitions required by national END implementation were adopted. In Italy, the day period corresponds to 06:00 to 20:00, the evening period to 20:00 to 22:00, and the night period to 22:00 to 06:00. In Romania, the day period spans 07:00 to 19:00, the evening period 19:00 to 23:00, and the night period 23:00 to 07:00. The second approach estimates short-term noise levels at a ten-minute resolution by combining CNOSSOS-EU sound power models with propagation matrices from commercial sound propagation software. Predicted levels were then compared with the in situ acoustic measurements collected at each test site, as illustrated in Figure 1, which shows the monitoring station equipped with the microwave radar traffic counter and the artificial intelligence-based camera used during the acquisition campaigns.

### 2.1. Test Sites

Three primary test sites were selected to ensure that the analysis covered peri-urban and urban conditions, different traffic regimes, and distinct built environments. One test site is located in Italy, and the other two are located in Romania. Table 1 summarizes the main geometric and operational characteristics of the three monitored road segments, including the number of lanes, the posted speed limit, and the distance between the road and the nearest residential façades. Distances are reported as the range of values measured at the receiver locations for each test site.

#### 2.1.1. Italian Test Site

**Figure 2:**
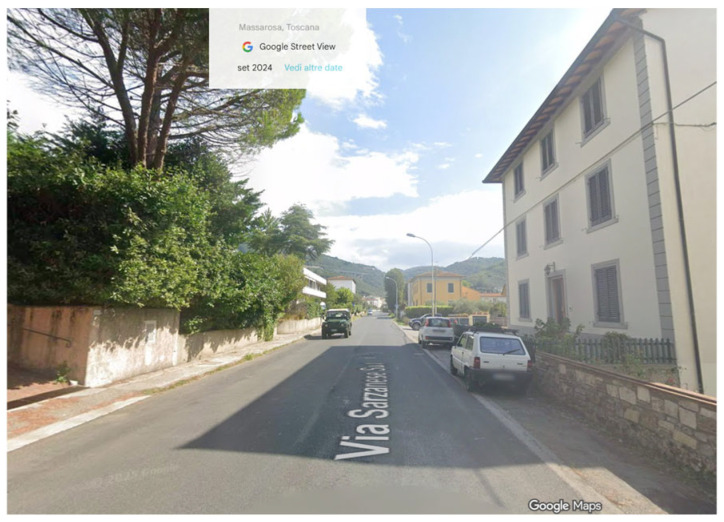
Italian test site. SR 439 (1).

The Italian test site, illustrated in Figure 2, is located along the regional road SR 439 in Massarosa (LU), Tuscany. The road crosses several small towns with approximately two thousand inhabitants. Most residential buildings are directly exposed to the road, and a primary school that was built in the early twentieth century and recently renovated is located near the monitored section.

Traffic patterns are characterized by pronounced peaks during morning and evening commuting hours. The vehicular fleet includes a significant proportion of light commercial vehicles and three-wheelers, which account for about 2% of the municipality’s fleet. Powered two-wheelers represent around 20% of all registered vehicles, reflecting the broader Italian context. This site was previously included in the LIFE NEREiDE project [36,37], where an experimental low-noise pavement containing crumb rubber was installed in 2018. Measurements showed a noise reduction of approximately 5 dB(A) just six months after the laying. The surrounding terrain is moderately hilly and was considered in the noise modeling.

#### 2.1.2. Romanian Test Sites

**Figure 3:**
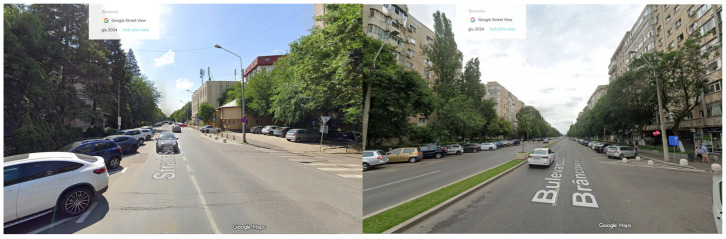
Romanian test sites. Left: Secuilor Street (2). Right: Constantin Brâncoveanu Boulevard (3).

The Romanian test sites illustrated in Figure 3 are located in District 4 of Bucharest, a densely populated and highly trafficked area of the capital. Bucharest has one of the slowest average traffic speeds among European capitals, with frequent congestion and significant delays (according to the TomTom traffic index 2024 [38]). District 4 spans approximately 34 km^2^ and includes residential buildings, schools, and a hospital. No railways or airports are located near the selected streets, although tram lines are present. Two roads, both in an urban context and flat terrain, were examined:

- Secuilor Street is a residential street with one lane in each direction and traffic volumes below five hundred vehicles per hour. Buildings along the way consist mainly of four-story residential blocks.
- Constantin Brâncoveanu Boulevard is a major connector with two lanes in each direction and traffic volumes between one thousand and fifteen hundred vehicles per hour. The boulevard is bordered by tall apartment buildings exceeding thirty meters in height.

### 2.2. Traffic Data Collection Methods

#### 2.2.1. Microwave Radar Traffic Counters (TC)

Microwave radar traffic counters were installed along the roadside to monitor up to two lanes. Sensors were placed either at approximately 0.5 m above ground for the Italian test site or at more than 2 m for the Romanian test sites. Higher placement reduces the likelihood of vehicle occlusion. Vehicle classification is based on measured length; therefore, calibration of the length thresholds is required. Radar counters cannot distinguish between lanes traveling in the same direction, and the detected vehicle length varies depending on the distance from the sensor, thus, on-site. Despite these limitations, they are easy to install, do not capture personal data, and allow simple and fast data processing. At test site 3, two TC devices were used (one per direction) to cover the four-lane boulevard.

#### 2.2.2. Artificial Intelligence-Based Cameras (CAM)

Artificial intelligence-based cameras equipped with deep learning vehicle detection, based on the YOLO approach to object detection [28], were used to classify vehicles according to CNOSSOS-EU categories. Up to four lanes can be monitored simultaneously, enabling accurate lane-specific flow determination. Speed estimation requires geometric calibration based on the distance between the camera and the lanes, which may require temporary closure of the road. Due to hardware constraints in the equipment used, post-processing of video streams was necessary to derive flows and speeds. In this scope, a future CAM version will include real-time output. Based on early tests and validations performed by the CAM provider, these cameras allow precise vehicle recognition but may overestimate flows when traffic volumes become very high.

#### 2.2.3. Google API-Derived Flows (API)

Google API-derived flows were obtained from travel time queries on selected origin –destination pairs. These crowdsourced data provide travel times for specific road segments, which can be used to estimate traffic volumes. Based on a basic transport equation, namely the Bureau of Public Roads (BPR) equation, a methodology was applied to convert travel times into passenger car equivalent flows using Python scripts [35]. The BPR equation was inverted to derive flow dependency from travel time (Equation (1)).

(1) $$F_{eq}=C*{\left(\frac{\frac{t}{t_{f}}-1}{\alpha }\right)}^{\frac{1}{\beta }}$$

where:

- $F_{eq}$ is the equivalent traffic flow;
- C is the road capacity;
- t is the travel time provided by the API;
- $t_{f}$ is the travel time when the road is free of traffic (inverse of free speed);
- α and β are transportation parameters of the road.

These flows were then allocated to CNOSSOS-EU vehicle categories based on modal split assumptions or standard percentages of vehicle categories when local information was not available. This method enables the remote acquisition of traffic information without on-site instrumentation and supports the creation of large datasets at fine temporal resolution. It is relevant to note that the API service always provides travel time values, even when the number of vehicles providing travel times is not sufficient to produce a sound estimation (including cases with no vehicles at all): in these cases, the Google API returns its last estimate without informing the user whether the estimate is based on currently available data or not, thus limiting users’ awareness of data reliability.

The approach has been shown to generate substantial and useful traffic datasets in contexts where no direct measurements are available. However, accuracy decreases under very low-flow or highly congested conditions because travel time becomes decoupled from the actual flow. The API approach, therefore, requires careful calibration of the underlying traffic model. It should also be noted that the Google API is not free of charge, and each origin–destination request has an associated cost of approximately USD 0.01.

### 2.3. Noise Measurements

Noise measurements were performed near the monitored road sections for approximately 24 h. Measurements for the Italian test site were conducted in April 2024, while measurements for both Romanian test sites were conducted in September 2024. Class 1 sound level meters were used in all locations, and instruments were mounted at a height of 4 m above ground level at roadside positions to ensure representative exposure conditions. Sound pressure levels were recorded continuously during the same periods in which traffic data were collected to enable a direct comparison between measured and predicted noise levels. Time histories of the acoustic signal were inspected manually to remove anomalous sound events such as sirens, horns, construction activities, or any other sources not related to traffic that could bias the analysis.

Time histories were manually inspected to remove short, clearly identifiable anomalous events unrelated to road traffic (e.g., sirens, horns). Exclusions typically involved a few seconds; if excluded portions exceeded 30% of an aggregation interval, that interval was discarded.

Meteorological data were also acquired throughout each campaign. Periods affected by rain or by wind speeds exceeding 5 m/s were excluded from the analysis, in accordance with established best practice for environmental noise measurements. While standard practice and local Italian regulations normally require at least a full week of road traffic noise measurements to infer long-term exposure indicators, this study was limited to observing differences in 24 h noise estimates derived from different input data collection methods without targeting reliable absolute L_DEN_ or L_N_ levels.

### 2.4. Procedures for Noise Simulation and Measured–Predicted Comparison

Point simulations were carried out in commercial noise modeling software at the receiver positions where noise measurements were performed. A separate scenario was created for each test site and for each traffic data collection method using identical geographic and propagation settings. In the Romanian urban context, only the two monitored streets were included in the simulations, even though other streets are present in the area. This choice was justified because it was initially assumed that the measurement positions were far enough from other major streets for their contribution to be negligible, and the local urban structure was expected to shield the receivers from traffic outside the monitored streets. In the Italian test site, the digital elevation model was included to account for the hilly terrain, whereas a flat terrain was used for the Bucharest test sites.

**Figure 4:**
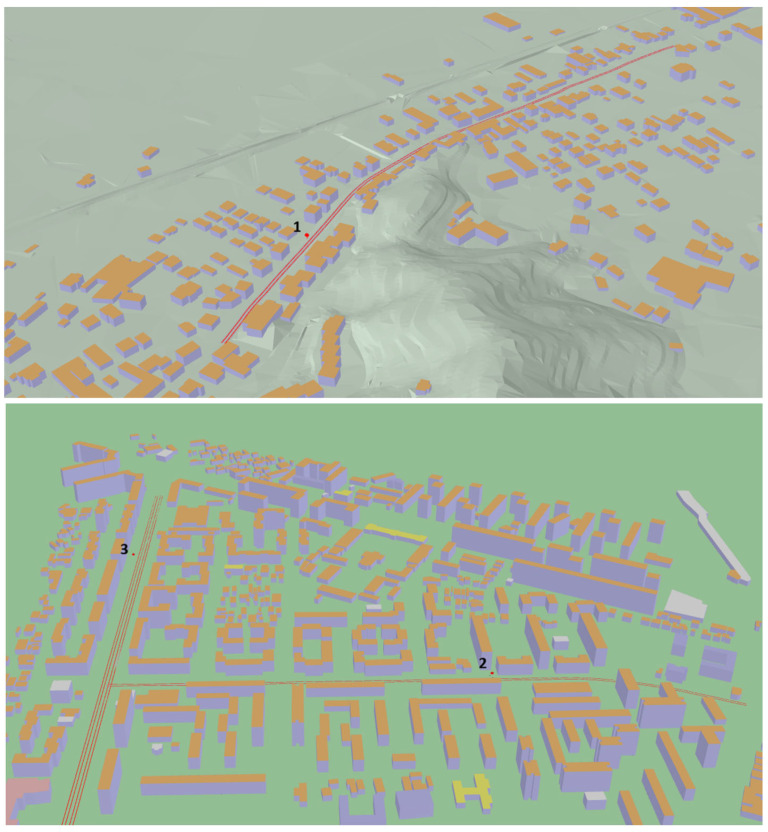
Representation of the test site areas as inserted into the 3D noise models. Above, the Italian test site (1), and below, the Romanian test sites (2) and (3).

Figure 4 shows the simulated scenarios with the distribution of source lines and receivers’ positions.

A dual approach was defined to enable complementary analyses. The first approach reproduces the standard END methodology and evaluates how different traffic data collection methods influence day, evening, and night indicators. The second approach evaluates the temporal variability of traffic inputs and their influence on noise levels at a higher time resolution. Together, the two approaches allow a comprehensive assessment of how microwave radar traffic counters, artificial intelligence-based cameras, and Google API-derived flows affect CNOSSOS-EU noise estimates under different temporal assumptions.

#### 2.4.1. First Approach

In the first approach, END noise mapping indicators for the day, evening, and night periods (L_D_, L_E_, L_N_, and L_DEN_) were computed using the CNOSSOS-EU model based on the average flows for light, medium, and heavy vehicles and powered two-wheelers derived from each traffic data collection method. Time slices followed the national definitions for each country. Noise levels, predicted using each input dataset, were then compared with the measured levels at the corresponding receivers.

This approach evaluates whether the average flows per time slice obtained from each method lead to accurate END indicators when used as inputs for the noise modeling software.

#### 2.4.2. Second Approach

The second analysis uses the ten-minute sampling rate of the traffic datasets to investigate differences between the input data collection methods at a finer temporal resolution. Running full noise simulations for each ten-minute interval would require excessive computational effort. Therefore, a method was developed to compute the noise levels from granular flows without rerunning the noise model, except for the derivation of attenuation matrices between sources and receivers. This procedure builds on the findings of the PRIN project OUTFIT [32] and consists of four steps.

Step 1. Sound power calculation

For each lane, vehicle category, and A-weighted octave band, the sound power level was calculated using the CNOSSOS-EU emission model with the flow and average speed measured in each ten-minute interval. This calculation was performed separately for each traffic data collection method by using Equation (2).

(2) $$L_{W^{\prime },eq,line,i,m}=10\log_{10}\left(10^{\frac{A_{R,i,m}+B_{R,i,m}\log_{10}\left(\frac{v_{m}}{v_{ref}}\right)}{10}}+10^{\frac{A_{P,i,m}+B_{P,i,m}\left(\frac{v_{m}-v_{ref}}{v_{ref}}\right)}{10}}\right)+10\log_{10}\left(\frac{Q_{m}}{v_{m}*1000}\right)$$

where:

- $A_{R,i,m}$, $B_{R,i,m}$, $A_{P,i,m}$, and $B_{P,i,m}$ are the CNOSSOS-EU coefficients for rolling and propulsion noise;
- $Q_{m}$ is the flow of the mth category;
- $v_{m}$ is the average speed of the mth category;
- $v_{ref}=70\;km/h$ is the reference speed in CNOSSOS-EU.

Step 2. Energetic summation

The total A-weighted sound power level for each lane was obtained by applying the energetic summation principle to the category-specific sound power levels (Equation (3)).

(3) $$L_{W\prime A,lane}=10*\log_{10}\left(\sum_{i,m}10^{0.1*\left(L_{W^{\prime },eq,line,i,m}+{AWC}_{i}\right)}\right)$$

where ${AWC}_{i}$ is the A-weighting correction for each frequency band.

Step 3. Propagation attenuation

Propagation attenuation from each lane to each receiver was derived from SoundPLAN with the same digital scenarios used in the first approach. SoundPLAN was used with the CNOSSOS-EU (2015) implementation and the same digital scenario and propagation settings adopted in the first approach. Consequently, any residual mismatch between the modeled and real propagation conditions would equally affect both approaches and does not impact the comparative analysis of the input methods.

A reference scenario with a flow of 10 vehicles per lane was simulated, and the resulting sound pressure levels at each receiver were used to derive the attenuation matrices by subtracting the lane-specific sound power level (Equation (4)).

(4) $$D_{receiver,lane}=L_{Aeq,receiver}-L_{W\prime A,lane}$$

where $L_{Aeq,receiver}$ is the sound pressure level computed in noise modeling software for the reference flow, and $L_{W\prime A,lane}$ is the corresponding lane sound power level.

Step 4. Reconstruction of sound pressure levels

For each ten-minute interval, sound pressure levels at the receiver positions were calculated by combining the lane-specific sound power levels with the corresponding attenuation matrices and summing the contributions energetically (Equation (5)).

(5) $$L_{Aeq,receiver}=10\;*\;\log_{10}\left(\sum_{lane}10^{0.1*\left({L_{W\prime A,lane}+D_{receiver,lane}}_{,}\right)}\right)$$

Day, evening, and night indicators were then computed from the energetic averaging of the reconstructed 10’ levels.

This methodological framework enables a consistent comparison between input data collection methods at both aggregated and short-term temporal scales.

### 2.5. Evaluation of the Traffic Data Collection Methods

The performance of the three traffic data collection methods was evaluated through a combination of quantitative and qualitative analyses. The quantitative assessment compared the traffic outputs of each method with reference values obtained from manual counting performed on selected 10’ intervals during daytime and evening periods and on one-hour intervals during nighttime. This comparison allowed the computation of traffic loss, defined as the percentage deviation between the manual counts and the flows derived from each method. Traffic loss was used to identify systematic under-detection or over-detection behaviors that may influence the accuracy of noise simulations. Additional traffic-based indicators included total flows, vehicle category distributions, and the temporal variability of flows at a 10’ resolution.

Manual counting was designed as a targeted diagnostic verification focused on periods with the largest discrepancies, not as a statistically representative sampling of the full 24 h traffic cycle. Therefore, the results from the manual counts should be interpreted as indicative of potential error mechanisms in challenging conditions rather than as certification of average method accuracy. A limited set of intervals was selected per site and period to ensure the feasibility of manual inspection while covering the most critical discrepancies.

Noise-based evaluation criteria were derived by comparing the measured acoustic levels with the simulated levels obtained using each traffic data collection method. The comparison used both aggregated END indicators (L_D_, L_E_, L_N_, and L_DEN_) and short-term reconstructed 10’ noise levels. The deviations between measured and simulated noise levels were expressed as ΔTC, ΔCAM, and ΔAPI, corresponding to the differences obtained when using radar counters, cameras, and API-derived flows as inputs. These indicators quantified the propagation of traffic estimation errors through the CNOSSOS-EU model and highlighted the sensitivity of noise predictions to the quality of the input data.

The analysis also examined the consistency of each method across the three test sites in order to identify recurring behaviors linked to traffic intensity, road geometry, and sensor deployment conditions. This cross-test site evaluation was essential for distinguishing context-dependent effects from method-dependent characteristics and for understanding whether observed discrepancies were related to traffic conditions or to the intrinsic properties of each method.

To complement the quantitative evaluation, a qualitative SWOT (Strengths, Weaknesses, Opportunities, and Threats) analysis was performed for each traffic data collection method. SWOT is a structured framework commonly used to synthesize the operational performance of alternative technologies, and its inclusion in this study provides an integrated perspective on the practical applicability of TC, CAM, and API by summarizing their advantages, limitations, external constraints, and potential for future development. The SWOT analysis, therefore, supports decision-making by identifying the conditions under which each method can realistically be used for road traffic noise monitoring and strategic noise mapping.

## 3. Results

The results are presented following the structure of the two modeling approaches described in Section 2.4. First, the analysis focuses on traffic inputs aggregated over the day, evening, and night periods, comparing total flows and the corresponding noise levels predicted with the END-based approach. This provides an initial assessment of how microwave radar traffic counters, artificial intelligence-based cameras, and Google API-derived flows differ when used to generate standard noise mapping indicators.

The second part of the analysis examines the traffic datasets at their native ten-minute resolution. Differences between the three input data collection methods are evaluated across time, followed by a comparison between measured and reconstructed ten-minute noise levels obtained with the second approach. This allows for an assessment of how short-term variability in traffic inputs propagates to noise estimates. The final part of the analysis computes day, evening, and night indicators that are derived from the second approach and compares them with the corresponding measurements.

### 3.1. Results of First Approach

In the first approach, the traffic flows aggregated over the day, evening, and night periods were used as inputs to the CNOSSOS-EU simulations. Table 2 reports the total flows estimated by the three input data collection methods in the three monitored test sites.

For the Italian test site, TC, CAM, and API provide comparable total flows during the day period, while differences become more pronounced in the evening and night periods. CAM remains consistent with TC across all periods. API, however, becomes unreliable when traffic volumes are low, because a single traffic model was applied to all time slices. For this reason, API data for the Italian test site are not considered further in the evening and night periods.

In the Romanian residential test site, the three methods show similar values during the day period. During the evening, TC tends to underestimate the total flow because the radar counter is less effective when traffic intensity decreases. API shows reduced sensitivity at low flows due to travel time smoothing and begins to diverge in the evening and night periods. The night period shows the largest variability among the three methods.

In the Romanian boulevard test site, the highest traffic volumes amplify the differences between the methods. TC systematically underestimates flows due to the position of the sensor, which caused a partial loss of vehicles in one of the two directions. CAM produces the highest and most stable estimates, reflecting its capacity to detect vehicles accurately, even in dense traffic. API shows large deviations, especially in the evening and night periods, because travel time-based estimation becomes unstable under highly congested conditions.

In all test sites, the percentage of heavy vehicles differs between TC and CAM (API is derived from CAM) because TC estimates heavy vehicles based on vehicle length, often confusing very narrow cars with heavy vehicles. The TC percentage values are significantly higher in the third test case, not because the absolute numbers differ substantially, but because the total number of vehicles is much lower than that estimated by CAM.

Using the flows divided by time slice and the corresponding average speeds, CNOSSOS-EU simulations were performed and compared with the measured levels. Table 3 reports the measured day, evening, and night noise levels and the deviations between the measured and simulated values obtained using TC, CAM, and API inputs (ΔTC, ΔCAM, and ΔAPI).

For the Italian test site, CAM and TC provide small deviations across all time periods, remaining within one decibel, while API was not applied in the evening and night periods due to the evident unreliability from traffic analysis.

In the Romanian residential test site, CAM provides the closest agreement with measured values during all periods. TC overestimates levels, reflecting the influence of vehicle categories assignment under low traffic conditions. API overestimates the night levels, which is consistent with the flow overestimations observed in Table 2.

For the Romanian boulevard test site, API shows the best overall agreement with measurements for all time periods. TC underestimates the levels, which is consistent with the known traffic loss. CAM presents the largest deviations in evening and night simulations because detection at night is more challenging.

Across the three test sites, CAM provides the most consistent and reliable input data for END-based simulations. TC performs adequately in moderate traffic but underestimates the flows in high densities or might mismatch vehicle categories in low-flow periods. API can provide reasonable estimates when traffic volumes are stable and sufficiently high, but it is not reliable in low-flow contexts or during congested periods.

### 3.2. Results of Second Approach

**Figure 5:**
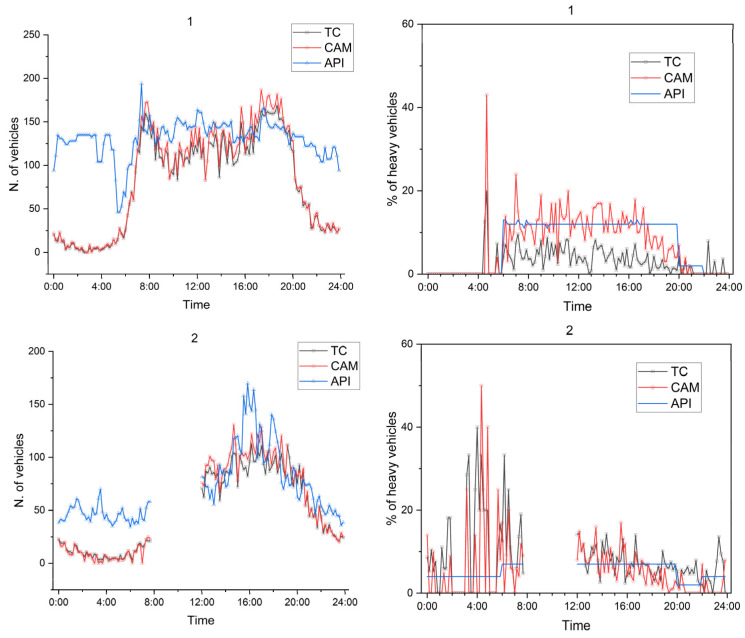

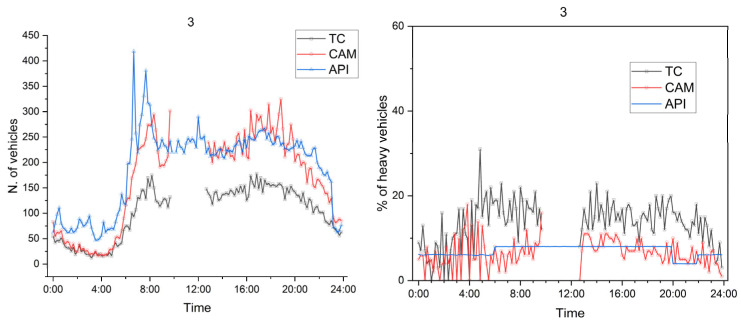
The 10’ total traffic flows and percentage of heavy vehicles for the three test sites, as derived from TC, CAM, and API.

A further analysis, based on greater temporal detail, was carried out using traffic flows and corresponding noise levels computed every 10’. This analysis compares the traffic estimates obtained from TC, CAM, and API in terms of the total flows and percentage of heavy vehicles. It must be noted that no prior information on vehicle categories was available before the measurements. Therefore, for the API approach, the modal split was assumed to match the average proportions derived from CAM for each time slice (day, evening, and night). Figure 5 reports the total flow and percentage of heavy vehicles for each test site.

TC and CAM exhibit comparable total flows, except at test site 3, which is consistent with the aggregated values observed in the first approach, although the percentage of heavy vehicles differs due to the distinct recognition principles of the two systems. At the Italian test site, it is more evident that the API produced unreliable evening and night traffic flows because the traffic model was not adjusted for those periods; namely, the parameters related to the transport equation used to transform travel times into flows (see Equation (1)) were maintained constant across the 24 h, while in Romania, different parameters were used for the three periods.

**Figure 6:**
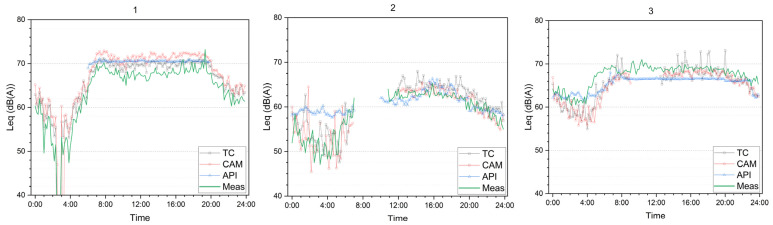
Measured and reconstructed 10’ noise levels values in the three test sites for the three input data collection methods (TC, CAM, and API) using the second approach.

These differences in short-term flow patterns directly affect the reconstructed noise levels. Figure 6 shows the noise levels estimated with the second approach and the corresponding measurements.

Fluctuations in the reconstructed noise levels are generally similar between measurements, TC, and CAM. In contrast, the API levels vary less and tend to remain above a threshold of approximately 55–60 dB at night, reflecting the fact that the API model performs adequately under continuous flow conditions but becomes unreliable for interrupted or low traffic flow.

In the Romanian boulevard test site, a “sunrise effect” was observed during the early morning period. Noise levels estimated from traffic inputs were approximately 5 dB lower than the measured levels, even though the flow differences were similar to those observed in other periods. This discrepancy is likely due to additional contributions from nearby markets and secondary streets; in fact, the differences in flows that might occur between real traffic and those measured by the input data collection methods cannot explain such a large difference. This interpretation was confirmed by inspection of the raw camera videos, as further discussed in the Discussion section.

**Figure 7:**
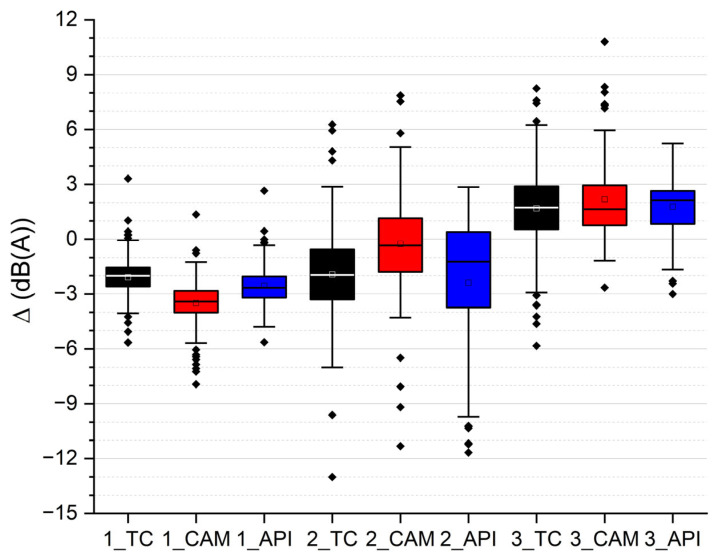
Box plot of differences between measured and reconstructed 10’ noise level values for each input data collection method (TC, CAM, and API) and test site.

The differences between measured and reconstructed noise levels for each input data collection method and test site are represented in Figure 7 as box plots of the whole dataset, while Table 4 summarizes the mean, median, and maximum absolute differences for each time period and for the full dataset. Although the 10 min series may exhibit temporal autocorrelation, methods are compared synchronously at identical timestamps; therefore, autocorrelation does not affect the comparative ranking among methods. Variability is assessed using robust descriptive indicators (median and maximum absolute deviation) together with box plots rather than inferential statistics, which is in line with the exploratory and operational scope of this study.

The statistics indicate that methods with mean differences close to zero may still exhibit large maximum deviations, which can influence longer-term averages.

In the Italian test site, all methods tend to overestimate the measured levels on average. This behavior is consistent with the presence of low-noise pavement at the site, which is not represented in the noise model and is estimated to reduce light-vehicle pass-by levels by at least 1 dB(A). Specific analyses are required to correctly model the pavement in the software. In this study, the specific pavement was modeled using the reference pavement, because six years after its laying, its residual functionality is expected to be low. The authors have developed a procedure to tune low-noise pavement coefficients in CNOSSOS-EU using pass-by data; however, the coefficients were established for this pavement only in the year following the laying [39]. To evaluate long-term coefficients, more data would be needed.

Although additional pass-by data suggest a residual benefit on the order of ~1 dB for light vehicles at the time of the campaign, a robust derivation of aged low-noise pavement coefficients for CNOSSOS-EU would require a dedicated study and is outside the scope of this work. The pavement assumption is kept identical across all input methods, so it does not affect the comparative ranking.

Based on this approach, average levels for L_D_, L_E_, and L_N_ were calculated and compared with the measurements. Table 5 reports on the measured values and the differences between measured and reconstructed levels for each input data collection method.

Considering the influence of the low-noise pavement at the Italian test site, only a few cases exceeded 3 dB differences: API, during the night in the residential test site, and TC and CAM during the night in the boulevard test site. Compared with the first approach, CAM shows slightly worse agreement during the night period because the sunrise effect produces a larger sound level difference than would be expected from the average flow alone. Despite this, CAM remains the most stable and accurate method among the three across all test sites.

Overall, the results of the second approach confirm that CAM provides the most consistent performance at the 10’ resolution, both in terms of temporal tracking and in the computation of L_D_, L_E_, and L_N_. TC performs reasonably well under moderate traffic but is affected by flow losses when traffic is dense and by category mismatches. API can offer useful flow estimates when traffic is stable, but its reliability decreases substantially under the traffic regimes that are typical of evening and night periods in both countries.

## 4. Discussion

The analyses carried out in this study provide a comprehensive comparison of the three input data collection methods across different traffic regimes and test sites. The results highlight clear patterns in the behavior of TC, CAM, and API, showing that the differences observed across the test sites depend not only on their geographical and operational environments but also on the progressive evolution of the methods themselves. The discussion examines these aspects in detail and includes an additional verification based on manual traffic counts performed over selected test periods, which supports the interpretation of the main findings and clarifies the practical challenges encountered during deployment.

The API method evolved between the Italian and Romanian campaigns, as it was refined in parallel through its application in the OUTFIT project [35]. The improvements focused on adjusting the traffic model parameters separately for each time period, which resulted in more accurate API-based traffic estimates during the Romanian measurements compared with those obtained in Italy. TC was deployed in all test sites, but the sensor position differed between countries because of specific safety requirements. In the Romanian boulevard test site, the higher mounting position caused a partial loss of detected flows. The CAM system was also progressively improved between the Italian and Romanian campaigns to reduce the impact of fog and humidity. Observed deviations are attributed primarily to operational conditions, including reduced illumination and headlight glare during evening and night periods, as well as occlusions under dense traffic conditions, and to the specific hardware version of the CAM system used during the campaign. These effects are related to field deployment constraints rather than to intrinsic limitations of the detection approach and do not affect the comparative conclusions of the study since the same CAM configuration was used consistently across all analyzed sites and periods.

Since all input data collection methods are affected by uncertainty, a focused comparison with manual counts was conducted using CAM raw videos. Manual counting was performed on selected intervals equal to 10 min for day and evening periods and one hour during nighttime. The validation presented in this paper represents a first verification of the observed data, and the use of 10 min intervals during the day and evening and 1 h intervals during the night was intended to ensure a significant number of vehicle passages; specifically, all periods and sites include at least 50 passages. The selection of intervals for comparison was made to target those in which the greatest differences with noise measurements were observed. Table 6 reports the resulting traffic losses, expressed as the percentage difference between the manual counts and the flows derived from each input data collection method. The percentage loss was computed, as in Equation (6).

(6) $$Loss\;\left(\%\right)=\frac{Manual\;count-Method\;estimate}{Manual\;count}\times 100$$

The results confirm that TC exhibits significant traffic loss for the boulevard test site, which is consistent with what was observed in the main analysis. API appears to be unreliable during nighttime, with large deviations linked to the instability of flow estimation under low traffic conditions. CAM generally provides the most accurate flow estimates, although it may overestimate flows or miss detections when traffic is very dense.

To further verify the consistency of the methods in estimating the category assignment, namely the percentage of light vehicles, Table 7 reports a comparison between manual counts and the values derived from each input data collection method. CAM aligns closely with manual counts across all test sites and time periods. TC tends to underestimate the percentage of light vehicles in several intervals, being that TC classifies several light vehicles that are too close to each other to be distinguished as heav vehicles. API shows variable performance depending on the time period and traffic conditions.

**Figure 8:**
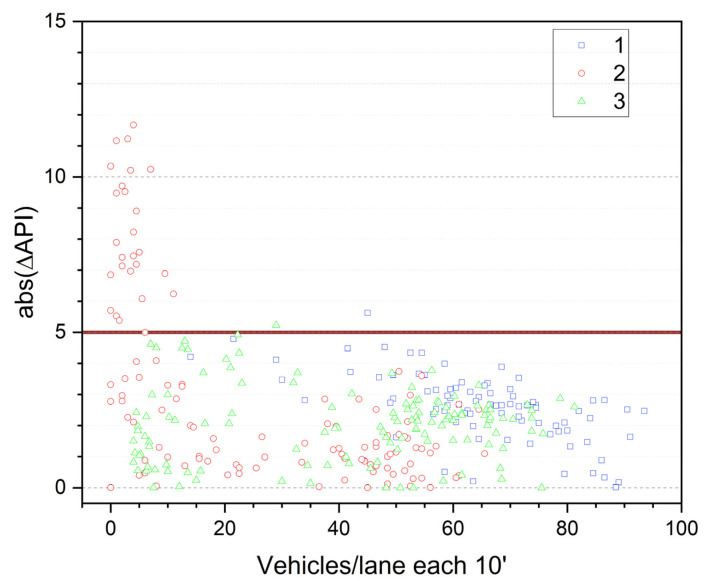
Difference between measured noise levels and API-based reconstructed levels (ΔAPI) as a function of traffic intensity, expressed as vehicles per lane per 10’.

A further analysis was carried out to investigate how the accuracy of the API approach varies with traffic intensity. Figure 8 shows the absolute difference between measured noise levels and those reconstructed using API inputs (ΔAPI), plotted against the number of vehicles per lane within each 10’ interval.

The analysis confirms that the accuracy of the API approach improves when traffic intensity is sufficient to influence travel times. When the flow exceeds approximately 25 vehicles per lane per 10 min (150 vehicles per hour), the error generally remains within 5 dB and appears more stable. This threshold is also related to the traffic model used, and thus to the application of the transport equation. Equivalent traffic flows derived by the equation change according to fleet composition, so the reliability threshold may also change. The identified threshold should be interpreted as an empirical operational indication for the investigated urban contexts (heavy-vehicle share <~10%) rather than a universal value.

The test sites all have proportions of heavy vehicles below approximately 10%, but if the proportion of heavy vehicles is higher, as for example on highways, fewer vehicles per hour may be required to obtain reliable data because this would correspond to a higher equivalent flow, which is generally more reliable than low traffic flows that are closer to free-flow conditions.

This analysis also suggests that below this threshold, the API should be complemented with other input methods. Default data based on local knowledge can be more reliable than API-derived inputs under these conditions. Further analyses may relate the flow-based threshold to the travel times returned by the API to verify whether the same value is provided for several consecutive intervals, suggesting that an insufficient number of vehicles is available to produce reliable travel time estimates.

The three input data collection methods also exhibit different performances depending on the objective and on the approach used to compute average day, evening, and night levels. Deriving equivalent levels from average flows, as in the first approach, is not equivalent to deriving the same indicators from noise levels computed for each 10’ interval, as in the second approach. To clarify how the two modeling strategies differ in practice, the indicators obtained from the first approach were directly compared with those derived from the 10’ reconstruction used in the second approach. Table 8 summarizes the differences between the two approaches for L_D_, L_E_, L_N_, and L_DEN_.

The two approaches differ because averaging traffic flows before sound power computation is not equivalent to averaging the sound levels computed at short time steps. Due to the logarithmic relationship between flow and level, short-term traffic peaks can contribute disproportionately to energetic averages. The 10 min reconstruction preserves this variability and thus provides additional insight into exposure patterns beyond standard long-term indicators [40,41].

The differences are largest in the Italian test site, which is located in a peri-urban area, and tend to be more pronounced during daytime. This reflects the non-linear relationship between flow and sound level and the sensitivity of the indicators to short-term fluctuations that are captured only in the second approach.

**Figure 9:**
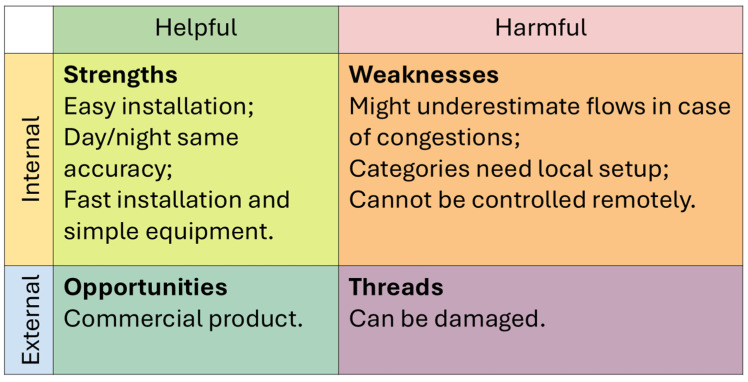
Qualitative SWOT analysis of the TC method based on the results obtained in the three test sites.

**Figure 10:**
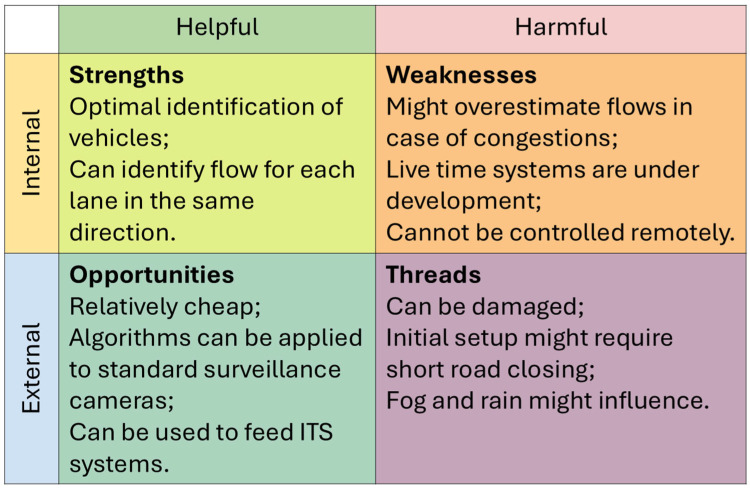
Qualitative SWOT analysis of the CAM method based on the results obtained in the three test sites.

**Figure 11:**
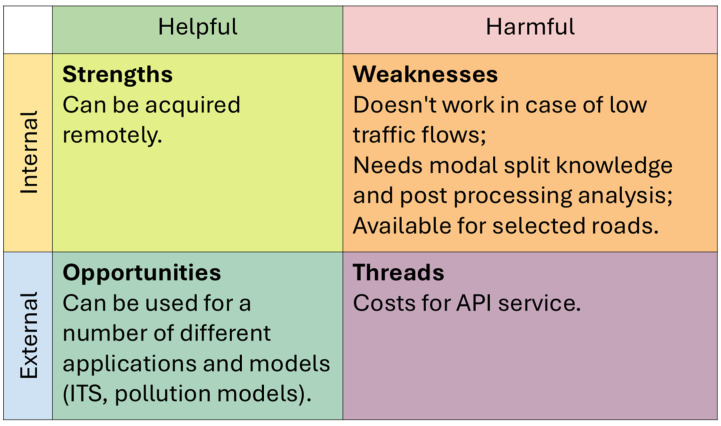
Qualitative SWOT analysis of the API method based on the results obtained in the three test sites.

All three input data collection methods present advantages and limitations. Figure 9, Figure 10 and Figure 11 summarize a qualitative SWOT analysis based on the outcomes of this study. This analysis is included to provide a practical synthesis of the operational implications of using TC, CAM, and API in real monitoring contexts.

TC is simple to deploy and stable across day and night, but it may underestimate flows during congestion. CAM provides accurate identification, lane separation, and consistent performance across the test sites, although its use may require temporary road closures to initialize the system, and it can be affected by adverse weather conditions. API has the advantage of being remotely accessible and applicable to different models, not only for noise, but its accuracy degrades significantly under low-flow conditions, and its use involves service costs.

Overall, the discussion confirms that CAM is the most reliable method across the different traffic regimes and test sites, that TC performs reasonably well under moderate traffic but suffers when vehicle detection becomes challenging, and API can provide useful information only when traffic volumes are sufficiently high to ensure stable flow estimation from travel times.

## 5. Conclusions

The present study evaluated the performance of three traffic data collection methods, namely microwave radar traffic counters (TC), artificial intelligence-based cameras (CAM), and Google API-derived flows (API), for use as inputs in road traffic noise modeling. The analyses were conducted across three test sites in Italy and Romania and were based on two complementary modeling strategies. The first approach used average day, evening, and night flows, while the second used reconstructed 10’ noise levels obtained from detailed flow profiles. The objective was to understand how different data sources affect the accuracy of noise predictions, as well as to identify the conditions under which each method can be reliably applied.

The results showed that TC and CAM provide consistent traffic estimates under most conditions, with CAM offering the closest alignment with measured noise levels across all test sites. TC performed well under moderate traffic, but it underestimated flows during congested periods. API produced acceptable estimates only when traffic was sufficiently high to influence travel times, and its performance degraded under low-intensity or unstable conditions. The differences between the two modeling approaches highlighted the importance of short-term traffic variability, particularly in peri-urban contexts and during the daytime. Manual counting confirmed the trends observed for each method and clarified the operational challenges encountered during field deployment.

This study advances the understanding of traffic input requirements for CNOSSOS-EU-based noise modeling. It demonstrates how methodological choices, sensor deployment conditions, and the evolution of the data collection techniques influence the accuracy of noise predictions. The inclusion of a qualitative SWOT analysis further supports the interpretation of the results by summarizing the operational strengths and weaknesses of each method and by providing practical guidance for their application in real monitoring scenarios. Moreover, the manual counting verification provided numerical evidence that all three input data collection methods exhibit non-negligible traffic loss. TC showed losses up to 23% in the Italian day period and 55% in the Romanian boulevard during the evening, while CAM displayed smaller deviations, generally within 5%, except for congested conditions, where it reached 37%. API showed the largest variability, with moderate deviations in some periods but extreme negative losses under low-flow or unstable conditions, such as minus 294% and minus 200% in the Romanian test sites during nighttime. These findings are consistent with reports in the literature showing that all commercial- and research-grade traffic sensors experience residual detection losses. Such losses directly propagate into noise simulations and tend to produce an underestimation of predicted traffic noise levels, seldom balanced by category mismatch, when used within CNOSSOS-EU-based models.

Several limitations must be acknowledged. The test sites represent only a subset of possible road environments, and the availability of modal split information influenced the reconstruction of API flows. Weather conditions affected CAM performance in specific periods, and the low-noise pavement in the Italian test site introduced differences that could not be fully captured by the model. The analysis of categories was constrained by the available data and was supported only by selected manual counting intervals.

Future developments should explore larger and more diverse test sites, refine the API modeling to better address low-flow conditions, and support real-time CAM systems to improve robustness under adverse weather. Progress in CAM technology is expected to further enhance performance, both through improvements in machine learning-based recognition algorithms and through advances in acquisition hardware that is designed to reduce the effects of humidity on the camera lens and to maintain stable operation under low illumination, taking advantage of infrared technology. Further work is also needed to integrate these data sources within decision support tools and to assess their potential in continuous monitoring frameworks and large-scale strategic noise mapping.

## Figures and Tables

**Table 1:** Characteristics of test sites.

Test Site	Country	Road Type	Lanes	Speed Limit (km/h)	Distance to Houses to Left and to Right Side (m)
1	IT	Regional	2	50	10-10
2	RO	Residential	2	30	15-20
3	RO	Boulevard	4	50	25-25

**Table 2:** Total traffic flows and percentage of heavy vehicles for the day, evening, and night periods in the three test sites, as derived from microwave radar traffic counters (TC), artificial intelligence-based cameras (CAM), and Google API-derived flows (API).

Test Site	Time Period	Number of Vehicles			% of Heavy Vehicles		
		TC	CAM	API	TC	CAM	API
1	Day	724	774	838	3.94%	11.29%	12.04%
1	Evening	365	423	769	1.37%	2.25%	1.69%
1	Night	81	96	692	1.24%	0.60%	0.00%
2	Day	472	558	504	7.48%	7.01%	7.00%
2	Evening	361	361	421	4.23%	1.81%	3.90%
2	Night	68	67	279	7.78%	5.67%	4.04%
3	Day	836	1471	1453	15.52%	7.74%	8.02%
3	Evening	724	1143	1265	14.44%	4.29%	5.40%
3	Night	254	355	595	11.67%	7.60%	6.72%

**Table 3:** Measured noise levels (dB(A)) for the day, evening, and night periods in the three test sites with the first approach. Deviations are reported as ΔTC, ΔCAM, and ΔAPI, corresponding to the differences between measured levels and CNOSSOS-EU predictions based on the TC, CAM, and API input flows.

Test Site	Metric	Measured Noise Level (dB(A))	Difference Measured–Predicted Noise Levels (dB(A))		
			ΔTC	ΔCAM	ΔAPI
1	L_D_	68.2	0.9	−0.6	0.1
1	L_E_	65.1	0.5	−0.7	--
1	L_N_	59.0	0.0	−1.0	--
1	L_DEN_	68.7	0.6	−0.8	--
2	L_D_	63.1	−0.8	−0.2	0.4
2	L_E_	60.3	−2.3	−0.3	−0.9
2	L_N_	54.6	−2.9	−1.2	−5.3
2	L_DEN_	64.0	−2.0	−0.6	−2.8
3	L_D_	69.1	2.5	1.2	1.7
3	L_E_	68.0	1.8	1.7	0.7
3	L_N_	65.5	3.2	2.4	0.4
3	L_DEN_	72.8	2.8	2.0	0.7

**Table 4:** Statistics of the differences between measured and reconstructed 10’ noise levels for each input data collection method, test site, and time period. In each cell, the first number is the mean, the second is the median, and the third is the maximum absolute difference.

Test Site	Time Period	Differences in Measured–Predicted Noise Levels (dB(A))		
		TC	CAM	API
1	Day	−1.7/−1.9/3.3	−3.4/−3.5/5.0	−2.5/−2.6/5.6
1	Evening	−1.8/−1.9/2.4	−2.7/−2.8/3.4	-
1	Night	−2.8/−2.6/5.7	−3.8/−3.4/7.9	-
1	24 h	−2.1/−2.0/5.7	−3.4/−3.4/7.9	-
2	Day	−2.1/−2.0/5.6	−0.7/−0.7/3.5	−0.4/−0.2/3.7
2	Evening	−1.8/−1.9/4.2	0.1/−0.2/3.1	0.1/0.1/2.9
2	Night	−1.9/−2.3/13.0	0.0/−0.1/11.3	−5.4/−5.5/11.7
2	24 h	−1.9/−1.9/13.0	−0.3/−0.3/11.3	−2.4/−1.2/11.7
3	Day	0.5/1.1/4.6	1.0/0.9/2.8	2.1/2.3/3.8
3	Evening	0.9/1.2/5.8	1.4/1.4/3.4	1.7/1.9/2.8
3	Night	3.3/3.4/8.2	3.8/3.5/10.8	1.5/1.7/5.2
3	24 h	1.7/1.7/8.2	2.2/1.6/10.8	1.8/2.1/5.2

**Table 5:** Measured noise levels (dB) for the day, evening, and night periods in the three test sites with the second approach. Deviations are reported as ΔTC, ΔCAM, and ΔAPI, corresponding to the differences between measured levels and CNOSSOS-EU predictions based on the TC, CAM, and API input flows.

Test Site	Metric	Measured Noise Level (dB(A))	Difference Measured–Predicted Noise Levels (dB(A))		
			ΔTC	ΔCAM	ΔAPI
1	L_D_	68.2	−1.6	−3.3	−2.3
1	L_E_	65.1	−1.8	−2.7	--
1	L_N_	59.0	−2.3	−3.2	--
1	L_DEN_	68.7	−2.0	−3.2	--
2	L_D_	63.1	−1.9	−0.5	−0.4
2	L_E_	60.3	−2.1	−0.1	0.2
2	L_N_	54.6	−2.0	−0.9	−4.3
2	L_DEN_	64.0	−2.0	−0.5	−2.2
3	L_D_	69.1	0.3	1.3	2.5
3	L_E_	68.0	0.6	1.3	1.7
3	L_N_	65.5	3.3	3.7	2.1
3	L_DEN_	72.8	2.0	2.6	2.1

**Table 6:** Percentage traffic loss for each input data collection method based on manual counts performed over selected intervals.

Test Site	Time Period	Percentage Traffic Loss		
		TC	CAM	API
1	Day (10’)	23%	−4%	−10%
1	Evening (10’)	7%	−1%	--
1	Night (1 h)	16%	−14%	--
2	Day (10’)	3%	−3%	18%
2	Evening (10’)	−4%	1%	−20%
2	Night (1 h)	7%	3%	−294%
3	Day (10’)	33%	−5%	−9%
3	Evening (10’)	55%	37%	27%
3	Night (1 h)	18%	4%	−200%

**Table 7:** Percentage of light vehicles derived from manual counts and from each input data collection method over selected intervals.

Test Site	Time Period	% of Light Vehicles			
		Manual Counts	TC	CAM	API
1	Day (10’)	86	83	82	80
1	Evening (10’)	96	93	97	--
1	Night (1 h)	92	82	94	--
2	Day (10’)	94	83	91	89
2	Evening (10’)	97	86	97	90
2	Night (1 h)	86	84	89	90
3	Day (10’)	87	84	87	90
3	Evening (10’)	90	76	88	92
3	Night (1 h)	88	87	89	92

**Table 8:** Differences between the first and the second approach for L_D_, L_E_, L_N_, and L_DEN_ in the three test sites.

Test Site	Metric	Difference Predicted 1st—Predicted 2nd Noise Levels (dB(A))		
		TC	CAM	API
1	L_D_	2.5	2.7	2.4
1	L_E_	2.3	2.0	--
1	L_N_	2.3	2.2	--
1	L_DEN_	2.5	2.4	--
2	L_D_	1.1	0.3	0.8
2	L_E_	−0.2	−0.2	−1.1
2	L_N_	−0.9	−0.4	−1.1
2	L_DEN_	0.0	−0.1	−0.7
3	L_D_	2.2	−0.1	−0.7
3	L_E_	1.2	0.3	−1.1
3	L_N_	−0.1	−1.3	−1.7
3	L_DEN_	−0.3	−0.6	−1.4

## Data Availability

The raw data supporting the conclusions of this article will be made available by the authors upon request.

## Abbreviations

The following abbreviations are used in this manuscript:

API	Google Application Programming Interface
CAM	Artificial intelligence-based camera
CNR	National Research Council of Italy
CNOSSOS-EU	Common Noise Assessment Methods in Europe
END	Environmental Noise Directive
ITS	Intelligent Transportation Systems
LD	Equivalent continuous sound level for the day period
LDEN	Day, evening, and night noise indicator
LE	Equivalent continuous sound level for the evening period
LN	Equivalent continuous sound level for the night period
TC	Microwave radar traffic counter
WHO	World Health Organization
